# Older Adults’ Awareness and Knowledge of Beans in Relation to Their Nutrient Content and Role in Chronic Disease Risk

**DOI:** 10.3390/nu11112680

**Published:** 2019-11-05

**Authors:** Katarina M. Doma, Emily L. Farrell, Erin R. Leith-Bailey, Victoria D. Soucier, Alison M. Duncan

**Affiliations:** Department of Human Health and Nutritional Sciences, University of Guelph, Guelph, ON N1G 2W1, Canada; kdoma@uoguelph.ca (K.M.D.); efarre01@uoguelph.ca (E.L.F.); eleithba@uoguelph.ca (E.R.L.-B.); vsoucier@uoguelph.ca (V.D.S.)

**Keywords:** nutrition awareness, beans, chronic disease risk, nutrition knowledge, older adults, mixed-methods, questionnaire, focus groups

## Abstract

Awareness and knowledge of nutrient-dense foods are important for older adults to help them make dietary choices that support a food-first approach to healthy aging. This is especially important since age is a major risk factor for chronic disease and the proportion of older adults in North America is increasing. Beans can contribute to a food-first approach to healthy aging as they are nutrient-dense and can reduce the risk of chronic diseases. However, studies exploring awareness and knowledge of beans in older adults are lacking. Therefore, the aim of this study was to explore older adults’ awareness of beans in relation to their nutrient content and role in chronic disease risk. Community-dwelling older adults (≥65 years old) were recruited and completed a validated researcher-administered questionnaire (*n* = 250), which was followed by 10 focus groups (*n* = 49). Results showed that the majority of older adults considered beans as a healthy food and thought consuming them could improve their health (99.2% and 98.0%, respectively); however, only 51.2% were bean consumers. While the majority (83.6%) of older adults were aware that a serving of beans is high in dietary fibre, bean consumers were significantly more likely to think that consuming beans could improve health areas related to dietary fibre including body weight management and constipation. Furthermore, most (84.8%) older adults thought consuming beans could improve heart health; however, bean consumers were significantly more likely to be aware that one serving of beans is low in nutrients relevant to heart health including total fat, saturated and *trans* fat as well as cholesterol. This research can help to inform healthcare professionals and public health agencies to create specific dietary strategies focusing on increasing older adults’ awareness and knowledge of beans in relation to their nutrient profile and role in promoting health.

## 1. Introduction

Awareness and knowledge of the nutrients that healthful foods provide and their potential to promote health can contribute to their increased consumption [1]. This is particularly important for older adults since the need for a nutrient-dense diet is enhanced by age-related declines in nutrient intake and absorption due to several factors. These factors include reduced appetite and food intake, changes in smell and taste, and medication interactions [2]. While most micronutrient requirements remain constant or increase with age, energy requirements decrease [3,4], therefore requiring less food intake which may also result in less nutrient intake. These factors could contribute to the 34% of Canadian [2] and 56.3% of American [5] older adults that are classified as being at nutritional risk. Thus, awareness and knowledge of nutrient-dense foods and their potential for health promotion and disease risk reduction can benefit older adults by better equipping them with dietary strategies to optimize healthy aging.

Beans are a nutrient-dense food that have been extensively studied for their health benefits. Beans contain high amounts of protein, complex carbohydrates and micronutrients, while remaining low in total fat, saturated and *trans* fat, and sodium and free of cholesterol [6,7]. Importantly, nutrients of concern for older adults such as dietary fibre and potassium are high in beans [8], providing rationale for the inclusion of beans in a healthful diet for older adults. The nutrient density of beans enables them to improve nutrient intake through the diet [9] and reduce the risk of chronic diseases such as cardiovascular disease, cancer and diabetes [10]; all of which older adults are at a higher risk of developing [8].

Improving the diets of older adults with nutrient-dense foods such as beans is particularly relevant since older adults are a rapidly growing proportion of the population, with the North American older adult population projected to increase to almost 30% by 2050 [11]. This demographic shift calls for a focus on educating older adults about nutrient-dense foods, such as beans, that can help reduce their risk of age-related chronic disease. However, research exploring awareness and knowledge of beans in relation to their nutrient profile and their role in health promotion is currently minimal. Therefore, the purpose of this study was to explore older adults’ awareness and knowledge of beans in relation to their nutrient profile and their potential to promote health through reducing chronic disease risk.

## 2. Materials and Methods

### 2.1. Participants and Study Design

Participants included older adults who were ≥65 years old, community-dwelling, English speaking and cognitively able to provide written consent. Recruitment occurred through flyers, advertisements on websites targeted towards older adults, and information tables at senior community centres and retirement villages. The study was advertised as a Food Survey Study to reduce participant bias with respect to bean consumption and a target of 250 older adults was established based on previous studies of similar design [12,13,14,15].

This study used a mixed-methods approach to allow for breadth (phase one: study questionnaire) and depth (phase two: focus groups) to the data collection. The study was approved by the University of Guelph Human Research Ethics Board (REB#16JL028) and all participants provided written consent before participation.

### 2.2. Phase One: Study Questionnaire

Phase one included a researcher-administered validated questionnaire that explored awareness of the nutrient composition of beans and their role in health and disease in relation to bean consumption status. The questionnaire was developed with reference to the literature and included open- and close-ended questions. The questionnaire was evaluated for content validity including clarity, representativeness and overall comprehensiveness [16]. The average overall comprehensiveness score was 3.92 out of 4 and the average inter-rater agreement (IRA) scores of 0.85 (clarity) and 0.96 (representativeness), and content validity indices (CVIs) of 1.0 were above acceptable values (0.7 for IRA scores [17] and 0.8 for CVI [18,19]). Reliability testing was completed using the test-retest method [20] with a success rate of 92% based on the average mismatch per question within each section of the questionnaire. Pilot testing of the questionnaire was completed in 20 older adults to ensure participant comprehension of the questions and to allow the research team to practice timing and flow of the questionnaire data collection.

The questionnaire was comprised of a variety of questions including current bean consumption frequency, which was identified as daily, weekly, monthly or never, and bean consumers were defined as those who consumed beans either daily or weekly. Participants were asked about their awareness of the amount of nutrients in one serving of canned beans using Health Canada’s daily values (DVs) as an indicator, wherein ≤5% of the DV is considered a low amount; 5.1–14.9% of the DV is considered a medium amount; and ≥15% of the DV is considered a high amount. Participants were also asked about beans in relation to Canada’s Food Guide (the 2007 version which was current at the time of data collection). Additional questions related to the nutrient quality of beans included identifying if beans have a low glycemic index, and identifying if the nutrient content differs between canned and dried, bagged beans; between rinsed and unrinsed canned beans; and among the different varieties of beans (a pictorial information document was provided that outlined the different bean varieties). Questions also explored whether participants thought bean consumption could improve health and which specific health areas bean consumption could improve. Lastly, medical, lifestyle and demographic information were collected. Data collection occurred at senior community centres, retirement villages, senior apartment buildings and the Human Nutraceutical Research Unit at the University of Guelph.

### 2.3. Phase Two: Focus Groups

Phase two involved ten focus groups, each consisting of 4 to 6 participants, which were completed at senior community centres and private boardrooms. Participants who completed the questionnaire were offered an opportunity to participate in the focus group phase. The research team completed focus group training to learn and implement focus group best practices [21]. Researcher roles were consistent and included the facilitator, who hosted the focus groups and generated discussion; the field note taker, who transcribed the focus groups during and immediately afterwards; the themes recorder, who observed and recorded key themes; and the attendant, who provided props throughout the focus groups. Props included a pictorial information document outlining the different bean varieties and flip chart paper to record comments and serve as a visual reminder of relevant comments to each question to help generate discussion. Each focus group was digitally audio-recorded and immediately transcribed verbatim and de-identified for qualitative data analysis purposes.

Focus group questions were constructed to explore participants’ awareness of beans in relation to their nutrient profile and contribution of those nutrients to health and chronic disease risk. Questions were developed using constructs of the social cognitive theory [22] and included “When you hear the term ‘beans’, which nutrients come to mind?” followed by “What health areas do you think these nutrients could improve?”. To ensure participant comprehension and appropriate timing and flow of facilitation, focus group questions were pilot tested in a sample of older adults (2 males, 1 female).

### 2.4. Data and Statistical Analysis

Statistical analysis was completed using the Statistical Analysis Software (SAS, version 9.4) with *p* ≤ 0.05 considered statistically significant. Prevalence of bean consumption was calculated as the proportion of participants who responded that they currently consume beans either daily or weekly, divided by the total number of participants. Questionnaire data were summarized using frequency and percentages for qualitative variables and mean ± SE for quantitative variables. Data were compared between bean consumers and bean non-consumers using the Chi-square test for qualitative data and an unpaired t-test for quantitative data. Logistic regression analyses were then conducted with the response variables as bean nutrients (dietary fibre, total fat, saturated and *trans* fat, cholesterol, iron, potassium, folate) and health areas related to beans (body weight management, constipation, diabetes, heart health), with the potential predictor variables as bean consumption status, medical and lifestyle variables (number of diagnosed health conditions, prescription medications and natural health products used, interest in overall health) and demographic variables (sex, education, marital status). Potential predictor variables that alone were moderately related (*p* < 0.10) to the response variable were included in the initial combined logistic regression models for each response variable. Model fit was then determined for each response variable and compared to a reduced model through a backward elimination approach using the likelihood ratio test to determine the best model fit.

Transcribed focus group records and themes documents were reviewed by the research team to ensure accuracy and consistency before import into NVivo11 Pro (©QSR International Pty Ltd., Melbourne, Australia) qualitative data analysis software. A word frequency query was completed to determine the most frequently mentioned nutrients that older adult participants identified as being present in beans. Words that were not relevant to the question or did not sufficiently represent the focus group discussions were assigned to the “Stop Words” list and removed from the query. Top nutrients were reviewed, and health areas related to these nutrients identified by older adults were used to generate themes. Themes were then reviewed and refined to ensure relevance to the research question.

## 3. Results

### 3.1. Participant Characteristics and Bean Consumption

Among the 250 older adult (≥65 years old) participants, 97.6% were white/European, 76.0% were female and 52.8% were married/common law and there were no significant differences between bean consumers and bean non-consumers (Table 1). The number of diagnosed health conditions, prescription medications and natural health products were 3.54 ± 0.14, 2.58 ± 0.14, 2.55 ± 0.13, respectively, again with no significant differences between bean consumers and bean non-consumers (Table 1). Prevalence of bean consumption was 51.2% with 128 older adults categorized as bean consumers (daily or weekly) and 122 older adults categorized as bean non-consumers (monthly or never).

### 3.2. Awareness of the Nutrient Content of Beans

The majority of older adults correctly thought that beans are a healthy food (99.2%) with no significant differences between bean consumers and bean non-consumers (100% vs. 98.4%; *p* = 0.20). Further, most bean consumers and bean non-consumers correctly thought that beans have a low glycemic index (81.3% vs. 80.3%; *p* = 0.50) and incorrectly thought that the nutritional composition does not vary among bean varieties (63.3% vs. 65.6%; *p* = 0.50) or between rinsed and unrinsed canned beans (74.2% vs. 68.9%; *p* = 0.30). However, bean consumers were significantly less likely than bean non-consumers to report that the nutritional composition varies between canned and dried, bagged beans (61.7% vs. 76.2%; *p* = 0.01).

The majority of all older adult participants (83.6%) correctly indicated that one serving of beans contains ≥15% DV of dietary fibre with no significant difference between bean consumers and bean non-consumers, and no predictor variables were significantly associated with the likelihood of this (data not shown). Dietary fibre was also one of the most frequently mentioned nutrients and was primarily related to digestive and bowel health by older adult participants in the focus group discussions.

The majority of older adult participants also correctly identified that beans contain ≤5% of the DV for total fat, saturated and *trans* fat, and cholesterol (72.8%, 75.6% and 66.8%, respectively); however, bean consumers were significantly more likely to do so (80.5% vs. 64.8%; *p* = 0.04 for total fat; 83.6% vs. 67.2%; *p* = 0.01 for saturated and *trans* fat; and 73.4% vs. 59.8%; *p* = 0.009 for cholesterol). The odds of older adults correctly identifying beans as containing ≤5% of the DV for total fat, saturated and *trans* fat, and cholesterol was related to being a bean consumer (OR = 2.49, 95% CI: 1.17–5.31, *p* = 0.02; OR = 3.28, 95% CI: 0.99–10.84, *p* = 0.052 and OR = 3.19, 95% CI: 1.39–7.33, *p* = 0.006, respectively) (Table 2). Focus group discussions also supported an emphasis on total fat wherein fat was one of the most frequently mentioned nutrients when participants heard the term ‘beans’.

Fewer participants were correct in identifying the micronutrient content of beans with only 36.4%, 17.6% and 17.2% of all participants correctly identifying that one serving of beans contains ≥15% DV for iron, potassium and folate, respectively, with no significant differences between bean consumers and bean non-consumers (data not shown). However, being female was significantly associated with the odds of participants correctly indicating the iron content of beans (OR = 2.22, 95% CI: 1.07–4.62, *p* = 0.03) (Table 2). Focus group discussions also revealed iron as one of the most frequently mentioned nutrients by older adults who related its presence in beans to the health of red blood cells. There were no predictor variables that were significantly related to older adults correctly indicating the potassium or folate content of beans (data not shown).

### 3.3. Awareness of the Relation of Beans to Health and Disease Risk

The majority of all older adult participants (98.0%) indicated that bean consumption could improve their health with no significant differences between bean consumers and bean non-consumers (99.2% and 96.7%; *p* = 0.16). More specifically, most older adults indicated bean consumption could improve body weight management, constipation, diabetes and heart health (86.4%, 94.0%, 78.8% and 84.8%, respectively). Bean consumers were significantly more likely than bean non-consumers to indicate that bean consumption could improve body weight management (92.2% vs. 80.3%; *p* = 0.006) and constipation (96.9% vs. 91.0%; *p* = 0.05) but not diabetes (83.6% vs. 73.8%; *p* = 0.058) or heart health (86.7% vs. 82.8%; *p* = 0.40). Predictor variables significantly associated with the identification of these health areas, beginning with body weight management, included being a bean consumer (OR = 2.89, 95% CI: 1.30–6.44, *p* = 0.009), being a university or college graduate or more (OR = 2.26, 95% CI: 1.05–4.84, *p* = 0.04) and the use of <3 natural health products (OR = 0.42, 95% CI: 0.19–0.94, *p* = 0.04); for constipation, being very interested in their overall health (OR = 7.31, 95% CI: 2.08–25.65, *p* = 0.002), number of diagnosed health conditions (OR = 4.67, 95% CI: 0.97–22.44, *p* = 0.054) and being a bean consumer (OR = 3.76, 95% CI: 1.10–12.93, *p* = 0.04); for diabetes, being married or common law (OR = 1.97, 95% CI: 1.06–3.65, *p* = 0.03); and for heart health, the use of <3 natural health products (OR = 0.32, 95% CI: 0.15–0.71, *p* = 0.005) (Table 3).

### 3.4. Awareness of Beans in Relation to Dietary Guidance

The majority of older adult participants were aware that beans are included in Canada’s Food Guide with no significant difference between bean consumers and bean non-consumers (87.5% vs. 81.2%; *p* = 0.20). Most participants thought beans were included in the correct meat and alternatives food group (53.9% vs. 53.3%; *p* = 0.97) followed by the incorrect fruit and vegetables (40.6% vs. 50.8%; *p* = 0.08), grain products (22.7% vs. 25.4%; *p* = 0.56) and milk and alternatives (1.6% vs. 1.6%; *p* = 0.95) groups, again with no significant differences between bean consumers and bean non-consumers.

## 4. Discussion

The current study explored older adults’ (≥65 years old) awareness of beans in relation to their nutrient profile and role in health promotion and disease prevention using a validated researcher-administered questionnaire followed by focus groups. This work adds to the limited literature [23,24,25,26] on awareness of the nutrition and human health properties of beans and can inform evidence-based dietary strategies to promote bean consumption and improve health in the rapidly growing demographic of older adults.

Prevalence of bean consumption in this sample of 250 older adults was only 51.2%, despite the fact that almost all participants agreed that beans are a healthy food and that bean consumption could improve their health. This discrepancy was also observed in an American study in which only 10.8%, 22.8% and 19% of low-income women (*n* = 158) reported bean consumption ≥5 times, 3–4 times and 1–2 times per week, respectively; yet the majority (69.6%) reported that beans could improve their nutrition [23]. The discrepancy between these positive health perceptions of beans and actual bean consumption could be due to identified barriers to bean consumption in older adults such as not thinking to include beans in meals, flatulence, or a lack of knowledge about preparing beans [24].

For beans in relation to their nutrient content, the majority of older adult participants correctly indicated that dietary fibre is high in one serving of beans. Although there were no predictor variables associated with this identification, bean consumers were significantly more likely to relate beans to improvement of health areas that dietary fibre can influence, including body weight management and constipation. A previous study of 177 older adults also found the majority (75%) to be aware that (baked) beans were a high source of fibre [25]. Dietary fibre is a core shortfall nutrient for older adults [8] and this work supports the idea that beans can provide a versatile solution.

The fact that beans are low in total fat, saturated and *trans* fat, and cholesterol was also correctly identified by the majority of older adult participants. However, bean consumers were more likely to be aware of the low content of these nutrients in beans and being a bean consumer was identified as a significant predictor of this awareness. These results are intuitive since more frequent consumption of a food is likely to prompt a greater familiarity with its nutrient content.

Although iron was one of the most frequently mentioned nutrients in focus group discussions, only about one third of older adults correctly indicated beans to be high in iron; therefore, calling out an opportunity for education. Females were more likely to indicate beans to be high in iron, which is logical since iron is a potential nutrient of concern for females [27] and females have been found to have greater nutrition knowledge relative to males [28]. Awareness of beans as a food to increase dietary iron was also documented in an American survey of primarily female Registered Dietitians (RDs) [26].

The potassium and folate content of beans was not as well understood, with the majority of participants indicating a lack of knowledge about their content in beans. This knowledge gap identifies an educational opportunity, particularly since RDs are aware of this, as shown in a survey of RDs (*n* = 296) in the US, where the majority indicated they think beans can increase dietary folate [26]. Potassium also deserves particular attention, since it has been identified as a shortfall nutrient in older adults [8], and beans could be a solution that has not previously been considered. Although protein was not investigated in the current study since it does not have a DV, its content is of interest as an option for older adults, particularly with the advance of protein foods in the revised 2019 Canada’s Food Guide [29]. Overall, these results regarding older adults’ awareness of the dietary fibre, fat, cholesterol, iron, potassium and folate content of beans can provide industry, public health agencies, and governmental agencies some insights on where and how to focus promotion of this nutrient-dense food.

For beans in relation to human health, almost all older adult participants thought that bean consumption could improve their health, which is encouraging and speaks to the potential for increased bean consumption in this demographic. The specific health areas that the majority of older adults thought bean consumption could improve included body weight management, constipation, diabetes and heart health. A focus on these health areas is consistent with a recent survey about beans (although in a different participant group of RDs; *n* = 296) in which the majority agreed or strongly agreed that beans could reduce constipation (51.5% and 43.7%, respectively), help with feelings of fullness, (25.5% and 73.1%, respectively), help to lose weight (59.4% and 28.3%, respectively), and lower low density lipoprotein (LDL) cholesterol (46.6% and 48.0%, respectively) [26]. In contrast, a study in low-income women (again a different participant group than the current study; *n* = 158) found that the majority of participants did not know bean consumption could “help lose weight” (52.9%) or “lower bad cholesterol” (56.7%); however, the majority agreed or strongly agreed (50.0% and 21.8%, respectively) that beans can “help you feel full” [23]. Nevertheless, body weight management, diabetes, digestive health and heart health align with the most commonly promoted health benefits of beans [30,31]. Further, predictor variables including bean consumption status and health and demographic variables (i.e., interest in overall health, number of diagnosed health conditions, number of natural health products, education and marital status), that the current study found to increase the likelihood of older adults identifying these health areas, provide rationale for tailoring the development of bean education programs.

Awareness of beans in relation to governmental dietary guidance was also examined in the current study, specifically using the 2007 Canada’s Food Guide which was current at the time of data collection. Most older adult participants were aware that beans were included in Canada’s Food Guide and, more specifically, in the meat and alternatives food group. These observations are consistent with a previous telephone survey study, wherein the majority of participants (≥18 years old) were “generally aware” of Canada’s Food Guide and, of those, the majority were “specifically aware” although no more detail was ascertained [32]. Similarly consistent is a questionnaire study completed in Swiss adults (50–80 years old) that found the majority were aware of the Swiss Food Pyramid [33]. While the majority of older adults in the current study were aware beans were included in Canada’s Food Guide and in the correct food group, it is important to note that some older adults indicated beans were included in other food groups including milk and alternatives, highlighting some confusion surrounding food classification. Taken together, these studies suggest that consumers are at least aware of dietary guidance resources. However, more research investigating the awareness of specific recommendations by older adults is critical to identify gaps that can be targeted with future dietary guidance campaigns that are appropriate for this demographic. Moreover, with the recent release of the revised 2019 Canada’s Food Guide [29], research exploring older adults’ awareness of beans as they are included therein is warranted to remain up to date on this topic.

Strengths of this study include the validity and reliability testing of the questionnaire, the researcher-administration of the questionnaire to ensure participant comprehension of each question, and the mixed-methods approach to data collection. Despite these strengths, there were also limitations including the researcher-administration of the questionnaire which could have potentially introduced interviewer bias, the self-reported data, and the lack of information on specific amounts of bean consumption. Furthermore, the high level of education and the lack of diversity in the participant sample compromises the generalizability of the study’s results.

## 5. Conclusions

In summary, beans are a nutrient-dense food that help reduce chronic disease risk; however, research regarding awareness of the nutrient content of beans and the role beans play in health and disease is lacking. The current study provides insight into the awareness of these topics in the rapidly growing, nutritionally vulnerable demographic of older adults. Results showed that most older adults are aware that beans provide a high amount of dietary fibre and are low in total fat, saturated and *trans* fat as well as cholesterol; however, they are less aware of the iron, potassium and folate content of beans. Older adults in this study reported thinking that bean consumption could improve their health with the majority indicating that beans could improve body weight management, constipation, diabetes and heart health. A variety of medical, lifestyle and demographic variables were identified as predictors of their awareness of the amounts of some nutrients in beans and the ability of bean consumption to improve different health areas. Lastly, most older adults were aware that beans are included in Canadian dietary recommendations. Results from this research can be utilized by a variety of stakeholders including public health agencies, industry, and healthcare professionals, as it highlights the gaps in the current awareness of the nutrient content of beans and their role in health and disease risk. These results are challenging to contextualize due to the lack of current evidence on these topics in general, but also in specific demographics, including older adults. Therefore, more research on this topic in a variety of demographic groups is warranted to contribute to evidence related to consumers’ awareness of the nutrient and health benefits of beans.

## Figures and Tables

**Table 1:** Participant demographic and health characteristics for community-dwelling older adults (≥65 years old) based on bean consumption status (*n* = 250) ^a^.

	Bean Consumers ^b^ (*n* = 128)	Bean Non-Consumers ^c^ (*n* = 122)
**Sex**		
Female	98 (76.6)	92 (75.4)
Male	30 (23.4)	30 (24.6)
**Age (years)**		
65–70	40 (31.3)	35 (28.7)
71–75	36 (28.1)	28 (22.9)
76–80	29 (22.7)	33 (27.1)
81–85	15 (11.7)	17 (13.9)
86 +	8 (6.3)	9 (7.4)
**Ethnicity**		
White/European	124 (96.9)	120 (98.4)
Non-White/Non-European ^d^	4 (3.1)	2 (1.6)
**Marital Status**		
Married/Common-Law	69 (53.9)	63 (51.6)
Divorced/Separated	21 (16.4)	20 (16.4)
Widowed	31 (24.2)	34 (27.9)
Single	7 (5.5)	5 (4.1)
**Education**		
Some high school	9 (7.0)	12 (9.8)
High school graduate	16 (12.5)	21 (17.2)
Some college or university	27 (21.1)	25 (20.5)
College or university graduate	57 (44.5)	39 (32.0)
Graduate/professional degree	19 (14.8)	25 (20.5)
**Employment Status**		
Employed	10 (7.8)	6 (4.9)
Retired	118 (92.2)	116 (95.1)
**Annual Household Income (Canadian $)**		
˂$25,000	18 (14.1)	19 (15.6)
$25,000–$49,999.99	43 (33.6)	34 (27.9)
$50,000–$74,999.99	24 (18.8)	25 (20.5)
$75,000–$99,999.99	21 (16.4)	25 (20.5)
$100,000+	11 (8.6)	9 (7.4)
Number of Diagnosed Health Conditions (mean ± SE)	3.6 ± 0.2	3.5 ± 0.2
Number of Prescription Medications (mean ± SE)	2.7 ± 0.2	2.4 ± 0.2
Number of Natural Health Products (mean ± SE)	2.5 ± 0.2	2.6 ± 0.2

^a^ Data are shown as *n* (%) unless indicated otherwise. Sample sizes may total < 250, as participants had the option of not answering a question. ^b^ Bean consumers were defined as those who consume beans on a daily or weekly basis. ^c^ Bean non-consumers were defined as those who consume beans on a monthly basis or never. ^d^ Includes Black/African/Caribbean, South East Asian, Aboriginal/First Nations/Metis.

**Table 2:** Predictor variables associated with older adults ≥65 years old (*n* = 250) indicating the correct vs. incorrect response for the amount of each nutrient in one serving of canned beans based on Health Canada’s daily values ^a,b,c^.

Variables	OR ^d^	95% CI ^e^	*p*-Value
**Total fat**			
Bean consumption status ^f,g^			
(bean consumers vs. bean non-consumers)	2.49	1.17–5.31	0.02
Education ^g,h^			
(university/college graduate or more vs. high school or less)	1.09	0.52–2.29	0.82
Interest in overall health ^f,h,i^			
(very interested vs. somewhat interested)	1.06	0.22–5.15	0.94
**Saturated and *trans* fat**			
Bean consumption status ^g^			
(bean consumers vs. bean non-consumers)	3.28	0.99–10.84	0.052
Interest in overall health ^h,i^			
(very interested vs. somewhat interested)	1.48	0.17–12.65	0.72
**Cholesterol**			
Bean consumption status ^f,g^			
(bean consumers vs. bean non-consumers)	3.19	1.39–7.33	0.006
Education ^g,h^			
(university/college graduate or more vs. high school or less)	1.90	0.87–4.14	0.11
Interest in overall health ^f,h,i^			
(very interested vs. somewhat interested)	1.22	0.24–6.20	0.81
**Iron**			
Sex ^g^			
(female vs. male)	2.22	1.07–4.62	0.03
Interest in overall health ^i,j^			
(very interested vs. somewhat interested)	1.96	0.49–7.92	0.34

^a^ Daily value (DV) ranges include low (≤5% of the DV), medium (5.1–14.9% of the DV), and high (≥15% of the DV). ^b^ Predictor variables included for each nutrient are those that contributed to the best model fit according to the likelihood ratio test. ^c^ Dietary fibre, potassium and folate did not have any significant predictors and are therefore, not included. ^d^ OR: Odds ratio. ^e^ CI: Confidence interval. Adjusted for ^f^ education, ^g^ interest in overall health, ^h^ bean consumption status, and ^j^ sex. ^i^ The reference category used was “somewhat interested”, as no participants indicated being “not interested” in their overall health.

**Table 3:** Predictor variables associated with older adults ≥65 years old (*n* = 250) indicating bean consumption could improve specific health areas ^a^.

Variables	OR b	95% CI c	*p*-Value
**Body Weight Management**			
Bean consumption status ^d,e^			
(bean consumers vs. bean non-consumers)	2.89	1.30–6.44	0.009
Education ^e,f^			
(university/college graduate or more vs. high school or less)	2.26	1.05–4.84	0.04
Number of natural health products ^d,f^			
(˂3 vs. ≥3)	0.42	0.19–0.94	0.04
**Constipation**			
Interest in overall health ^f,g,h^			
(very interested vs. somewhat interested)	7.31	2.08–25.65	0.002
Number of diagnosed health conditions ^f,i^			
(<3 vs. ≥3)	4.67	0.97–22.44	0.054
Bean consumption status ^h,i^			
(bean consumers vs. bean non-consumers)	3.76	1.10–12.93	0.04
**Diabetes**			
Marital status			
(married/common-law vs. single ^j^)	1.97	1.06–3.65	0.03
**Heart Disease**			
Number of natural health products			
(˂3 vs. ≥3)	0.32	0.15–0.71	0.005

^a^ Predictor variables included for each nutrient are those that contributed to the best model fit according to the likelihood ratio test. ^b^ OR: Odds ratio. ^c^ CI: Confidence interval. Adjusted for ^d^ education, ^e^ number of natural health products, ^f^ bean consumption status, ^h^ number of diagnosed health conditions, and ^i^ interest in overall health. ^g^ The reference category used was “somewhat interested” as no participants indicated being “not interested” in their overall health. ^j^ Includes divorced/separated, widowed, and never married.
